# Supplementary material for: Uncovering the microbiome of invasive sympatric European brown hares and European rabbits in Australia
Source: PeerJ. 2020 Aug 18;8:e9564. doi: 10.7717/peerj.9564 (PMC7441920; doi:10.7717/peerj.9564)
Supplement: Supplemental Information 1 [file peerj-08-9564-s001.docx]

**Supplemental Table S1: Metadata for hare and rabbit samples used in this study**

| **Sample** | **Sample name** | **Sex*** | **Weight (g)^** | **Collection month** | **Lactation status*** |
| --- | --- | --- | --- | --- | --- |
| Hare 1 | MF-136 | NR | NA | January | No |
| Hare 2 | MF-148 | F | 3100 | May | No |
| Hare 3 | MF-149 | M | 3700 | May | NA |
| Hare 4 | MF-150 | M | 3040 | June | NA |
| Hare 5 | MF-151 | M | 2920 | June | NA |
| Hare 6 | MF-152 | F | 3460 | June | No |
| Hare 7 | MF-155 | F | 3250 | July | No |
| Hare 8 | MF-156 | F | 3350 | July | No |
| Hare 9 | MF-157 | F | 4900 | September | Pregnant, lactating |
| Rabbit 1 | MF-138 | F | NA | February | Pregnant, lactating |
| Rabbit 2 | MF-139 | M | 1080 | March | NA |
| Rabbit 3 | MF-140 | F | 960 | March | No |
| Rabbit 4 | MF-141 | F | 1380 | March | Yes |
| Rabbit 5 | MF-142 | F | 1440 | March | Yes |
| Rabbit 6 | MF-143 | NR | 400^ | March | No |
| Rabbit 7 | MF-144 | M | 1400 | March | NA |
| Rabbit 8 | MF-145 | F | 1500 | March | Pregnant, lactating |
| Rabbit 9 | MF-146 | F | NA | April | Pregnant, lactating |
| Rabbit 10 | MF-147 | F | NA | April | No |
| Rabbit 11 | MF-153 | M | 1500 | June | NA |
| Rabbit 12 | MF-154 | M | 1400 | June | NA |

***** F female; M male; NR not recorded; NA not applicable

^ young rabbit (<12 weeks old)
