# Supplementary figures and images for: Uncovering the microbiome of invasive sympatric European brown hares and European rabbits in Australia
Source: PeerJ. 2020 Aug 18;8:e9564. doi: 10.7717/peerj.9564 (PMC7441920; doi:10.7717/peerj.9564)

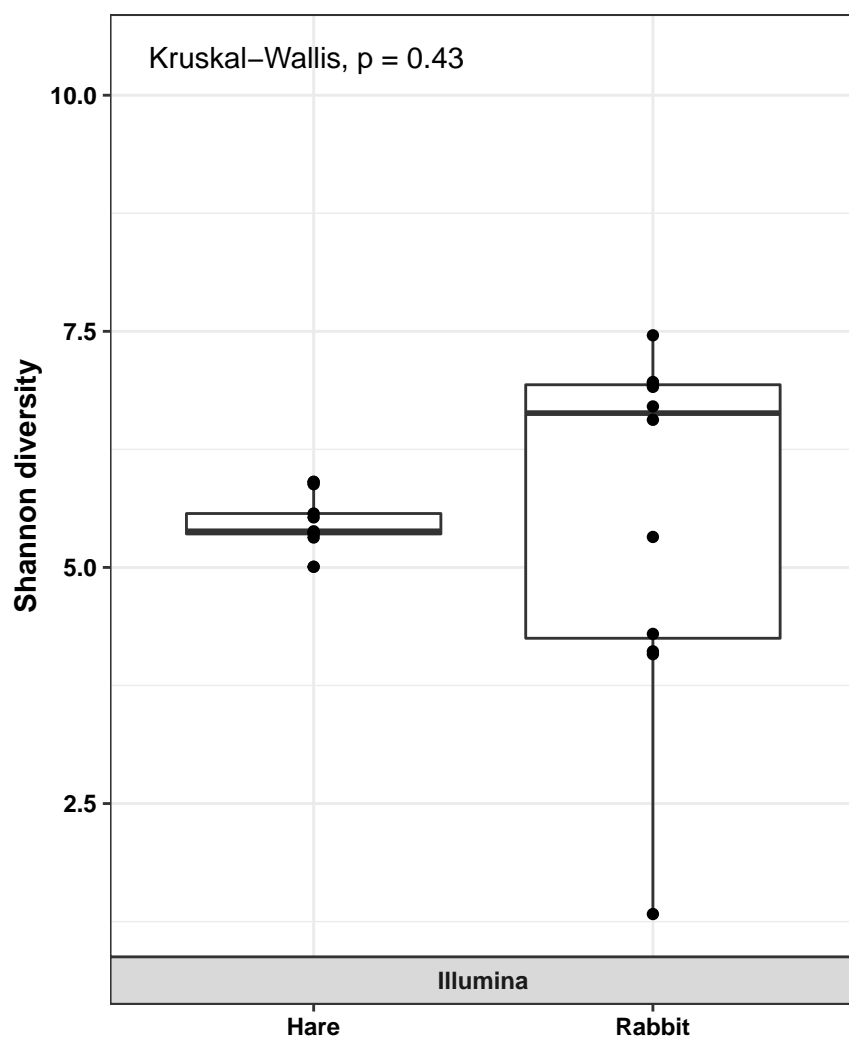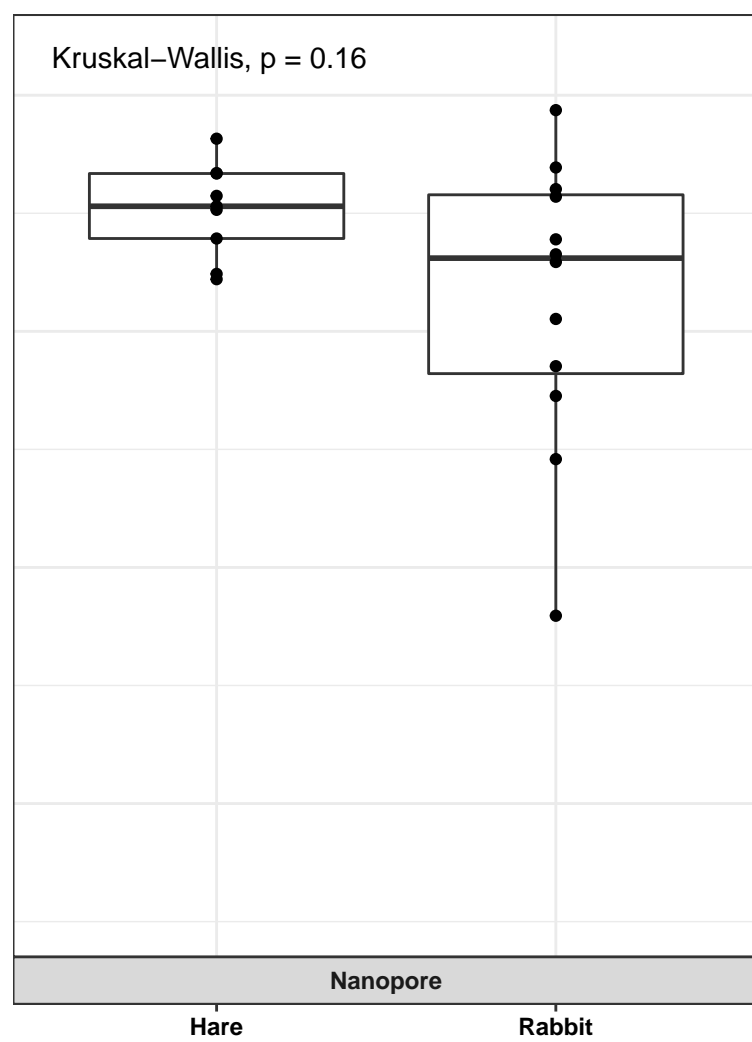

Supplement: Supplemental Information 3 — The richness and evenness of bacterial species in individual animals (alpha diversity) was estimated using the Shannon index calculated in QIIME2, and was compared between rabbits and hares using the Kruskal–Wallis test as implemented in the R package ‘ggpubr’. [file peerj-08-9564-s003.pdf]

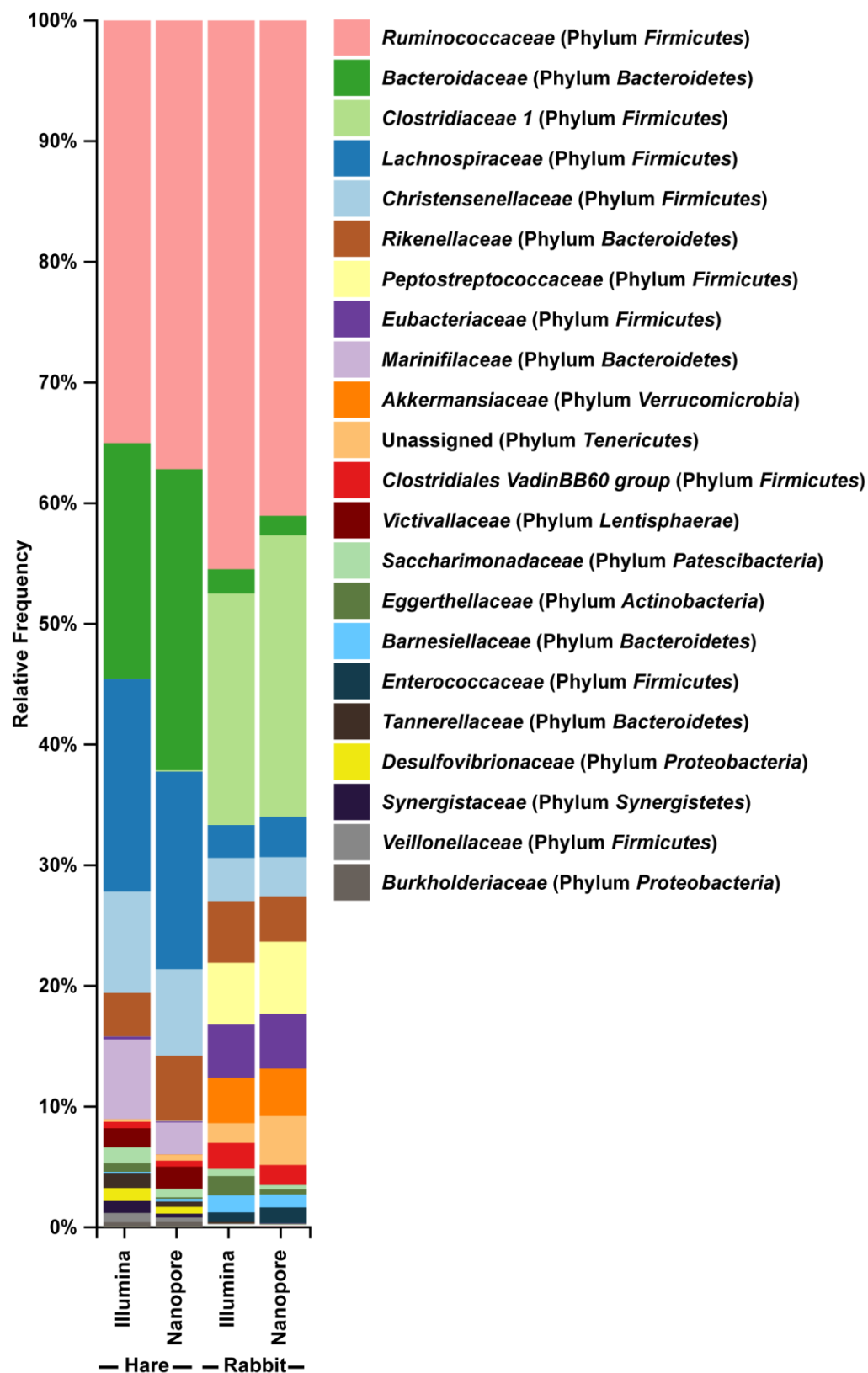

Supplement: Supplemental Information 4 — Taxonomic classification of 16S rRNA sequences from (A) Illumina and (B) Nanopore sequencing platforms was performed by alignment to the SILVA_132 reference database using BLAST+ within QIIME2 for Illumina data or BLASTn for Nanopore data. Results were combined for each host species (i.e. hare or rabbit) for each platform. Bacterial families present at a relative frequency less than 0.5% are not included. [file peerj-08-9564-s004.pdf]

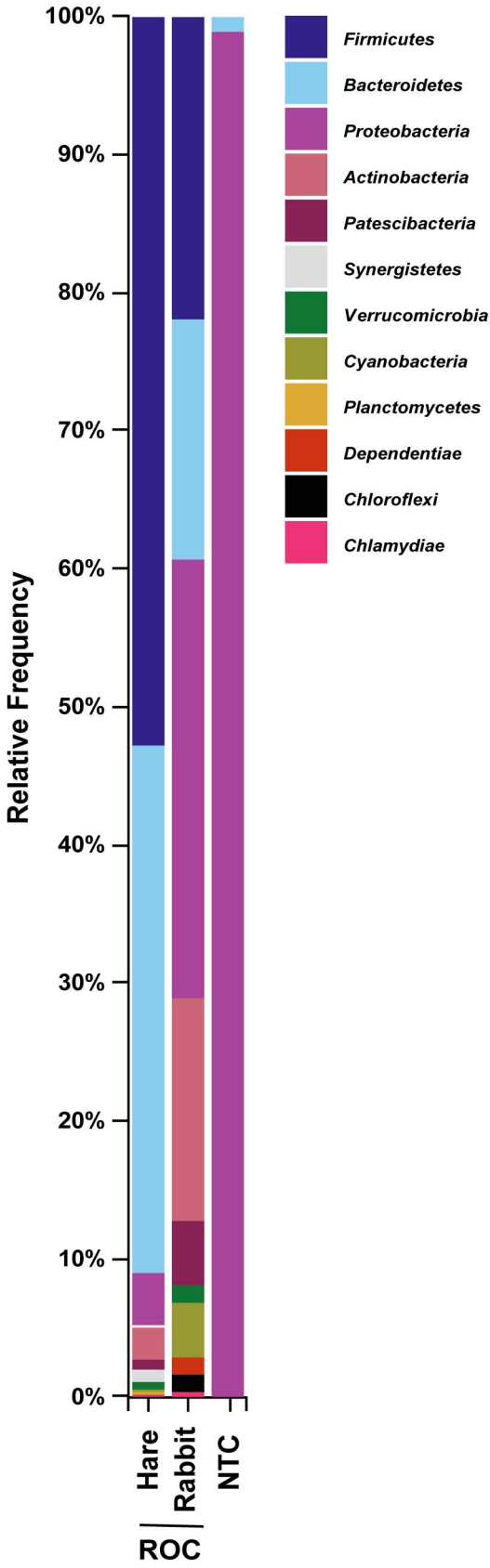

Supplement: Supplemental Information 5 — Reagent-only controls (ROCs) were extracted and processed in parallel with genomic DNA from faecal pellets. The no template control (NTC) was included in 16S rRNA PCRs and subsequently processed in parallel with samples. Samples were sequenced using the Illumina platform. Taxonomic classification of reads was performed using BLAST+ as implemented in QIIME2 against the SILVA_132 reference database. Bacterial phyla present at a relative frequency less than 0.5% are not included. [file peerj-08-9564-s005.pdf]
